# Supplementary material for: Cluster-based human-in-the-loop strategy for improving machine learning-based circulating tumor cell detection in liquid biopsy
Source: Patterns (N Y). 2025 May 30;6(6):101285. doi: 10.1016/j.patter.2025.101285 (PMC12191740; doi:10.1016/j.patter.2025.101285)
Supplement: Document S1. Figure S1 and Table S1 [file mmc1.pdf]

Patterns, Volume *6*

Supplemental information

Cluster-based human-in-the-loop strategy for improving machine learning-based circulating tumor cell detection in liquid biopsy

Hümeyra Husseini-Wüsthoff, Sabine Riethdorf, Andreas Schneeweiss, Andreas Trumpp, Klaus Pantel, Harriet Wikman, Maximilian Nielsen, and René Werner

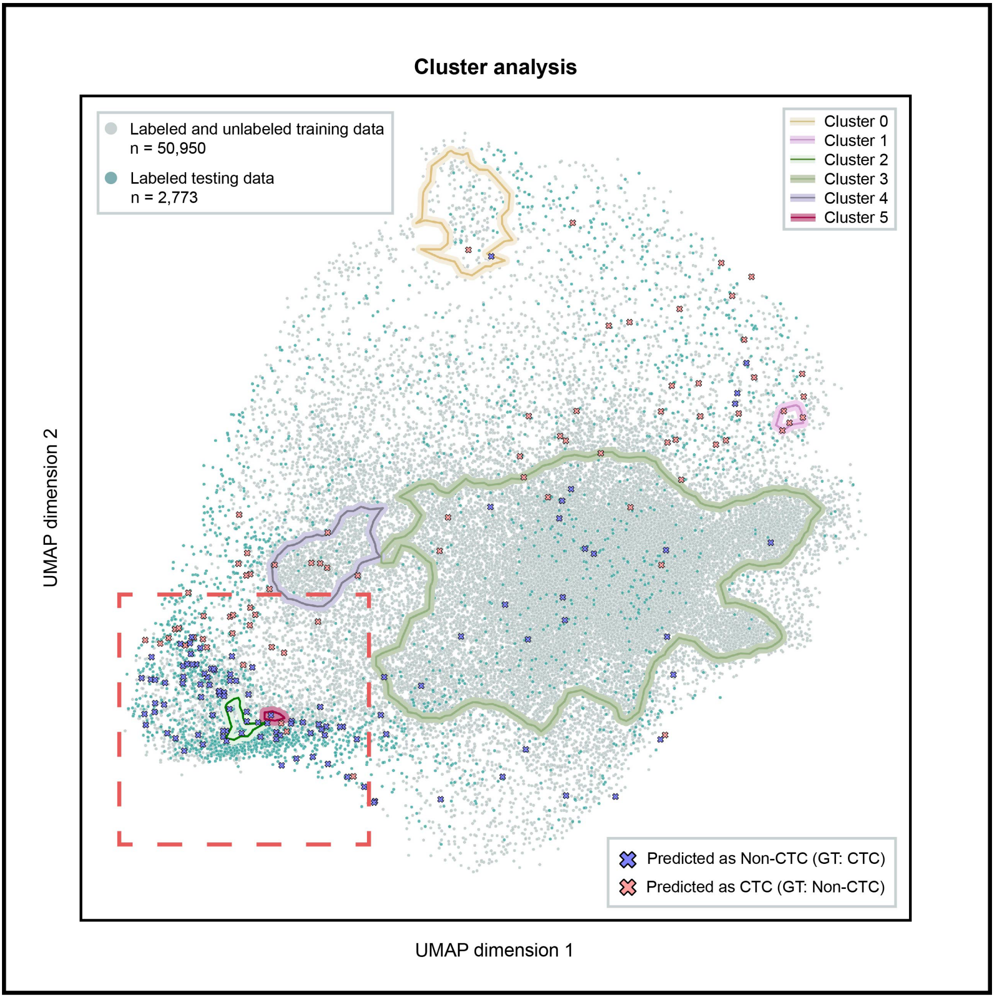

Figure S1: Distribution of clusters, related to Figure 2. Clustering was performed on the UMAP features of the joint labeled and unlabeled training data. Identified clusters are highlighted by closed contours. Data points not assigned to any of the identified cluster are defined as background. The red dashed box highlights the area where most test misclassifications occur, an area largely not captured effectively by any cluster. Abbreviations and explanations: GT: Ground truth.

Table S1: Comparison of machine learning model performance on the labeled test data, with and without hyperparameter optimization. The final loop of the "Simulated HiL scenario 2: limited local data" experiment was repeated across all 5 runs, substituting SVM with KNN and LR. Bayesian optimization of the hyperparameters with search spaces was performed separately for each run. For KNN, the optimized hyperparameters included n_neighbors, weights, metric, and algorithm. For LR, the hyperparameter C was optimized. For SVM, the hyperparameters C and gamma were optimized. Baseline refers to the model configuration with the default parameters from scikit-learn. The mean F1 score of each approach (for each ML model) across all runs was calculated on the complete labeled test set. Abbreviations: LR: Logistic regression; KNN: K-Nearest Neighbors; Opt.: Optimization.

ML model	Approach	Mean total F1 test score
KNN	Baseline	0.878
KNN	Bayesian Opt.	0.886
KNN	Proposed by Nielsen et al.[S1]	0.887
LR	Baseline	0.907
LR	Bayesian Opt.	0.909
LR	Proposed by Nielsen et al. [S1]	0.909
SVM	Baseline	0.924
SVM	Bayesian Opt.	0.921
SVM	Proposed by Nielsen et al. [S1]	0.921

Supplemental references

1. Nielsen, M., Wenderoth, L., Sentker, T., and Werner, R. (2023). Self-Supervision for Medical Image Classification: State-of-the-Art Performance with ~100 Labeled Training Samples per Class. Bioengineering (Basel) 10, 895. 10.3390/bioengineering10080895
